# Improved reperfusion following alternative surgical approach for experimental stroke in mice

**DOI:** 10.12688/f1000research.22594.3

**Published:** 2020-05-13

**Authors:** Melissa Trotman-Lucas, Raymond Wong, Stuart M. Allan, Claire L. Gibson

**Affiliations:** 1 School of Psychology, University of Nottingham, Nottingham, NG7 2UH, UK; 2 Faculty of Biology, Medicine and Health, University of Manchester, Manchester, UK

**Keywords:** Ischemia, Cerebral stroke, Reperfusion, Cerebral blood flow

## Abstract

**Background**: Following ischemic stroke, recanalisation and restoration of blood flow to the affected area of the brain is critical and directly correlates with patient recovery. *In vivo *models of ischemic stroke show high variability in outcomes, which may be due to variability in reperfusion.  We previously reported that a surgical refinement in the middle cerebral artery occlusion (MCAO) model of stroke, via repair of the common carotid artery (CCA), removes the reliance on the Circle of Willis for reperfusion and reduced infarct variability.  Here we further assess this refined surgical approach on reperfusion characteristics following transient MCAO in mice.

**Methods**: Mice underwent 60 min of MCAO, followed by either CCA repair or ligation at reperfusion.  All mice underwent laser speckle contrast imaging at baseline, 24 h and 48 h post-MCAO.

**Results**: CCA ligation reduced cerebral perfusion in the ipsilateral hemisphere compared to baseline (102.3 ± 4.57%) at 24 h (85.13 ± 16.09%; P < 0.01) and 48 h (75.04 ± 12.954%; P < 0.001) post-MCAO. Repair of the CCA returned perfusion to baseline (94.152 ± 2.44%) levels and perfusion was significantly improved compared to CCA ligation at both 24 h (102.83 ± 8.41%; P < 0.05) and 48 h (102.13 ± 9.34%; P < 0.001) post-MCAO.

**Conclusions**: Our findings show CCA repair, an alternative surgical approach for MCAO, results in improved ischemic hemisphere perfusion during the acute phase.

## Introduction

Cerebral ischemic stroke is one of the leading causes of death worldwide, carrying a significant disease burden as the second-leading cause of disability-adjusted life years ^1, 2^. Current approved treatment options are limited for acute ischemic stroke patients, involving either thrombolytic clot breakdown or physical clot removal. Recombinant tissue plasminogen activator (rtPA) is the only pharmacological treatment available with proven efficacy ^3, 4^, eligibility for this treatment is low and, due to a narrow therapeutic time window (<4.5 h), only ~15% of stroke patients are able to receive iv-rtPA treatment, with recanalisation success at less than 50% ^5, 6^. In addition to this, endovascular thrombectomy is increasingly being used in the treatment of large vessel occlusions, where the clot is removed from within the vessel allowing recanalisation, especially in patients who respond poorly to rtPA treatment ^5^. Prompt restoration of blood flow to the ischemic zone is the primary clinical goal, potentially salvaging tissue and preventing the spread of ischemic damage ^7^. Recanalization of occluded vessels is positively correlated with improved survival rates and recovery outcomes for ischemic stroke patients ^8^.

To allow development of new clinical therapeutics for stroke, the pathophysiology and mechanisms of disease/recovery need to be elucidated. Preclinical stroke models that closely mimic mechanisms of injury (and recovery) allow investigation of potential clinically viable treatments. Although experimental stroke models have shown the positive benefits of various potential therapeutics, these have repeatedly failed during clinical trials, preventing their progression into the clinic. This continued lack of translation requires us to further refine preclinical studies and to allow the evolution of more representative models ^9, 10^.

The most commonly used experimental model of ischemic stroke is the intraluminal filament model of middle cerebral artery occlusion (MCAO). First developed by Koizumi *et al.* ^11^ and later modified by Longa *et al.* ^12^, with numerous minor variations to the model since then. These two models are widely used in preclinical experimental stroke research largely due to their minimally invasive technique and ability to induce post-occlusion reperfusion ^13^. However, although surgical procedures follow a defined protocol, lesion volumes have large standard deviations ^14– 16^. With the Koizumi method, permanent ligation of the common carotid artery (CCA) is required, preventing return of flow through the internal carotid artery (ICA), thus reperfusion is dependent on collateral flow via the Circle of Willis (CoW). However, the CoW in C57BL/6J mice, commonly used in preclinical stroke models, is anatomically variable which affects collateral flow ability ^17, 18^. In addition to this, there are adverse consequences to animal wellbeing when impacting the external carotid artery (ECA), either for vessel entry as in the Longa modified model or for surgical ease as seen in the original Koizumi model, such as lick impairment affecting ability to drink alongside increased weight loss ^19^.

Stroke in the territory of the middle cerebral artery is the most common presentation seen in patients ^20^, thus targeting this vessel in preclinical models is valid. However, clot lysis induced by rtPA treatment results in gradual reperfusion to the ischemic territory, whereas in the MCAO model, filament removal is sudden giving an immediate blood flow surge to the ischemic area ^21^. In the clinic, endovascular thrombectomy allows physical removal of large vessel clots ^21, 22^, permitting rapid reflow through the previously occluded vessel, which is the primary clinical goal and parallels that which occurs in transient MCAO upon filament withdrawal. However, MCAO models where intraluminal access is obtained via the CCA, such as the Koizumi method ^23^, rely on collateral perfusion after filament removal due to the CCA being permanently tied off to prevent blood loss. Previously, we reported a modification to the Koizumi model of intraluminal filament MCAO, which allows bilateral CCA reperfusion, negating the reliance on the CoW ^10^. In commonly used mice strains such as the C57BL/6J, the variability seen in lesion volume may be largely due to anatomical variations in the CoW ^17^. Removing reperfusion reliance on the CoW reduces the variability seen in lesion volume but the impact on reperfusion is not known. Our aim here therefore is to determine if the adapted MCAO model can improve reperfusion following removal of the filament.

## Methods

### Animals

This study was conducted in accordance with the UK Animals (Scientific Procedures) Act, 1986, under Project license P28AA2253 following ethical approval from the University of Manchester. Animals were group housed (n=4) on arrival in temperature- and humidity-controlled individually ventilated mouse cages (IVCs) with a 12-h light/dark cycle, situated within a specific pathogen free (SPF) facility. Cages contained: woodchip bedding, paper sizzle nesting and cardboard tube. Animals were given *adlib* access to dry pellet food and water. Experimental sample size was determined using a power calculation from preliminary data (one tailed, α=0.001, β=0.9), taking into account a mortality rate of 10% established from historical MCAO data within our group. A total of 18 adult male C57BL/6J mice (10 weeks old on arrival; Charles River, UK), weighing 22–33 g at the time of MCAO, were used during this study: two animals were excluded due to surgical complications, two due to reaching humane endpoints and one due to repeated wound complications. All remaining animals (n=13) were humanely killed by cervical dislocation under anaesthesia following the 48-h post-MCAO scanning procedure. All experiments are reported in accordance with the Animal Research: Reporting of In Vivo Experiments (ARRIVE) guidelines 2018 ^24^.

### Study design

Individual animals were randomly assigned using an automated list randomiser ( random.org) to one of two experimental groups: CCA ligation or CCA repair. The CCA ligation group underwent 60 minutes transient MCAO with permanent CCA ligation following reperfusion ^25^. The CCA repair group underwent 60 minutes transient MCAO followed by repair of the CCA using a tissue pad and sealant combination, resulting in a patent CCA ^10^. Both groups underwent laser speckle contrast imaging (LSCI). The order of coded LSCI raw data files was randomised prior to analysis using an automated list randomiser. The experimenter was blind to groups during LSCI scanning and image analysis for raw data, data was decoded for statistical analysis only.

### Laser speckle contrast imaging

**Figure 1.**
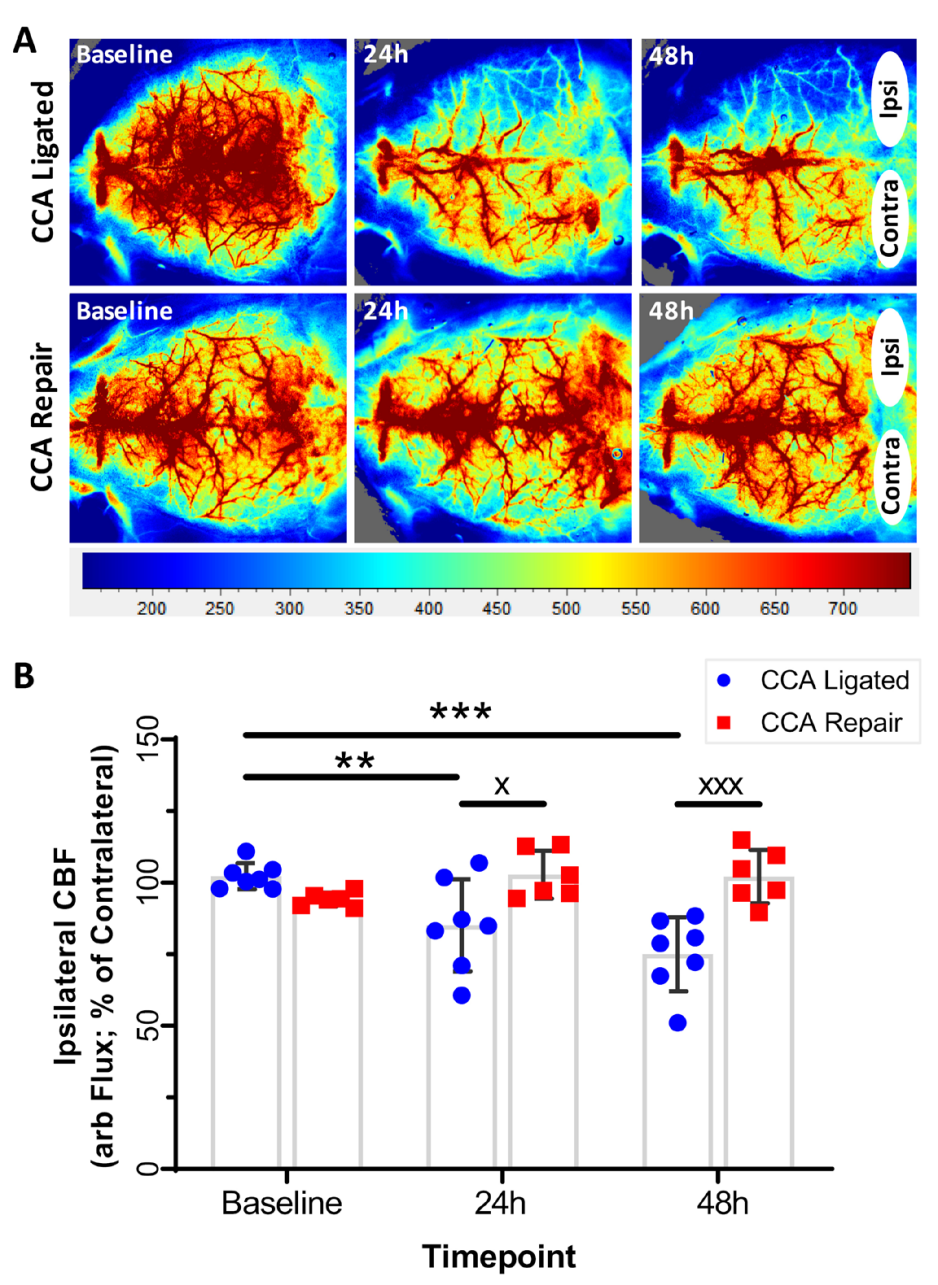
Cerebral blood flow measurements. Common carotid artery (CCA) vessel repair promotes cerebral blood flow (CBF) recovery following transient middle cerebral artery occlusion (MCAO). Regions of interest (ROIs) surrounding the ipsilateral and contralateral hemisphere were used to quantify CBF. ( **A**) Representative LSCI images at baseline, 24 h and 48 h post-MCAO in CCA Ligated and CCA repair mice. Contra, contralateral hemisphere; Ipsi, ipsilateral hemisphere. ( **B**) CBF obtained by LSCI, measured separately at baseline, 24 h and 48 h post-MCAO, expressed as ipsilateral flux as a percentage of contralateral flux. Statistical analysis performed using two-way ANOVA followed by Sidak post-hoc (ns *P* ≥ 0.05, */ ^X^ *P* ≤ 0.05, **/ ^XX^ *P* ≤ 0.01, ***/ ^XXX^ *P* ≤ 0.001). *Represents significance within experimental group versus Baseline. ^X^Represents significance between experimental groups. All data presented as mean ± SD (CCA Ligated *n*=7, CCA Repair *n*=6).

All animals underwent LSCI. Imaging was undertaken 12 days prior to MCAO (baseline imaging), then at 24 h and 48 h post-MCAO ( Figure 1A). Anaesthesia was induced and maintained with isoflurane (induction 5% in 100% O _2_; maintenance 1.5% in N _2_O/O _2_, 70/30%), following which mice were placed in a stereotaxic frame on a heat mat set to 37°C. The animals were positioned underneath a moorFLPI _2_ Full-Field Perfusion Imager (Moor Instruments, UK). Prior to incision Bupivacaine hydrochloride (2 mg/kg, Marcain 0.25%, AstraZeneca, UK) local anaesthesia was injected subcutaneous around the intended incision site. A midline incision was made and skin retracted to expose the intact skull. Ultrasound gel was applied to the skull, followed by a 10-mm ^2^ glass coverslip pressed down slightly to allow complete coverage of the intended scan area. LSCI was conducted for 3 min at 19 frames (20 ms per frame, 10-s intervals). The incision was cleaned free of ultrasound gel using sterile swabs and flushed with sterile saline, the incision was sutured using subcutaneous 6-0 dissolvable sutures followed by tissue adhesive application (Vetbond, US). The animal was returned to a pre-warmed clean cage for recovery. Following baseline LSCI only, animals were given access to paracetamol (200 mg/kg/24 h; Calpol®, Johnson & Johnson Ltd UK) in jelly for 24 h for self-administration analgesia. LSCI images were analysed using moorFLPI2 Full-Field Laser Perfusion Imager Review v5.0 software. For each animal, ipsilateral and contralateral hemisphere ROIs were drawn, ROI flux data was obtained from each hemisphere for each of the 19 images per scan, the mean of these values was used in later analyses. Ipsilateral hemisphere cerebral blood flow (CBF) is expressed as a percentage of the contralateral hemisphere CBF, within each imaging session. All analysis was conducted blinded to experimental conditions.

### Surgical procedure

All mice underwent transient MCAO as previously detailed, and surgical methods used were identical to those published previously by the author ^10, 26^, modified only to exclude Laser Doppler Flowmetry recording at MCAO reperfusion. MCAO was confirmed using laser doppler flowmetry (LDF), distinguished as a significant sustained drop in LDF flux once the microfilament filament was advanced into the internal carotid artery.

### Statistical analysis

All data are expressed as mean ± standard deviation of the mean (SD). All statistical analysis was performed using Prism v8 for windows (GraphPad Software, CA). The criterion for statistical significance was *P* ≤ 0.05. Distribution of data was assessed using Shapiro-Wilk normality test, prior to statistical analysis. Repeated measure two-way analysis of variance (ANOVA) test was used to determine effect of time and intervention, repair and ligation. Post-hoc Sidak corrected multiple comparison testing was then used to compare within groups against baseline/pre-MCAO, and within time point between groups.

## Results

There was a significant interaction between the two factors, time and CCA incision closure method/intervention, suggesting the effect of CCA intervention on CBF differs between the timepoints (F _(2,22)_ = 17, P < 0.001; Figure 1B)). CCA intervention post-MCAO, either ligation or repair, significantly affected ipsilateral CBF perfusion overall (F _(1,11)_ = 7.4, P = 0.02). Mice that underwent CCA ligation showed significantly reduced ipsilateral CBF compared to baseline (102.3 ± 4.57%) at 24 h (85.13 ± 16.09%; P = 0.001) and 48 h (75.04 ± 12.954%; P < 0.001) post-MCAO. Contrastingly, mice that underwent CCA repair showed no difference versus baseline (94.152 ± 2.44%) at 24 h (102.83 ± 8.41%; P = 0.191) or 48 h (102.13 ± 9.34%; P = 0.251) post-MCAO. There was no difference in ipsilateral CBF perfusion between groups at baseline (P = 0.417). At both 24 h and 48 h post-MCAO ipsilateral CBF was significantly increased in CCA repair mice compared to CCA ligated mice (P = 0.0122, P = 0.0001 respectively).

## Discussion

Here we report CCA vessel repair following MCAO in mice results in improved reperfusion of the ischemic hemisphere, assessed using LSCI. Ligation of the CCA following MCAO filament removal significantly reduced perfusion to the ischemic hemisphere for a prolonged period post-surgery and the CBF did not return to baseline up to 48 h post-MCAO. Repair of the CCA incision, using a homologous tissue pad and TISSEEL sealant as previously reported ^10^, returned hemispheric perfusion to pre-MCAO baseline levels within 24 h post-MCAO. The use of CCA repair in mice has been shown to improve immediate MCA territory blood flow within 5 minutes following repair and reduce lesion volume variability at 48 h post-MCAO ^10^. Data here show the initial post-MCAO return to baseline CBF, as shown previously, is sustained during the acute phase (i.e. 48 h) with no evidence of hypo or hyperperfusion in the ipsilateral hemisphere at 24 and 48 h post-MCAO.

The data indicate CCA repair improves ischemic area perfusion post-MCAO, we assume this is due to the return to patency of the CCA removing the reliance on collateral flow through the CoW. The CoW has been shown in C57BL/6J mice to be anatomically variable across individual animals ^17^, adding variability to collateral supply to the ischemic area. Similarly, Smith *et al.* reported, using MCA territory laser doppler flowmetry to measure perfusion, significantly increased post-MCAO perfusion using the Longa ECA entry method with perfusion returning to comparable baseline levels, whereas use of the Koizumi CCA entry method showed only a return to 50% of baseline levels ^23^. The key difference between the two methods is that the Longa method allows reperfusion through the bilateral CCAs whereas the Koizumi method relies on collateral supply to perfuse the ischemic area due to only the CCA being patent. These results mimic those reported here with CCA repair resulting in bilaterally patent CCAs leading to a return to baseline in perfusion post-MCAO. The use of a muscle pad combined with a fibrin sealant was previously shown in rat MCAO to allow forward blood flow through the CCA with complete occlusion of the vessel not present following the procedure ^27^. Additionally, the CCA repair method ensures that the ECA remains in situ and patent, reducing the impact on animal welfare and recovery ^19^.

Allowing reperfusion to occur through all available vessels transforms the MCAO model to be more representative of clinical endovascular thrombectomy. Endovascular thrombectomy is employed in an increasing number of clinical cases of large vessel occlusion (LVO), allowing mechanical, within vessel, removal of the occluding clot leading to a surge reperfusion of the ischemic zone, such as is seen following MCAO filament removal ^21^. There is discussion in the literature as to the relevance of MCAO for modelling clinical stroke, due to the slow gradual clot dispersion that occurs following rtPA treatment, a mechanism unlike the sudden increase in flow seen at MCAO filament removal ^28^, with suggestion that this slower clinical rtPA treatment reperfusion profile is closer to other embolic stroke models ^21, 29^. However, due to the positive effects of using endovascular thrombectomy for ischemic stroke treatment, on blood flow and patient outcome, the intervention is likely to become the primary treatment for LVO ^21^. With this in mind, there is renewed interest in the intraluminal filament model of MCAO as an established model of endovascular thrombectomy.

The data presented here suggest a beneficial effect on post-MCAO reperfusion in the ischemic hemisphere however, the limitations of our approach must be considered. LSCI has very good spatial and temporal resolution and is used to monitor CBF changes under both physiological and pathophysiological conditions but it may be that the low penetration depth of the laser does not permit measurements of deep brain CBF but only allows imaging of surface and pial vessels ^30^. This limits the interpretation of the data presented here, in terms of deep level reperfusion and further investigation would be required to assess the ability of CCA repair to improve deep level perfusion. In addition to this limitation, cerebral perfusion data is typically reported as raw arbitrary units or normalised to a baseline recording ^31, 32^, whereas the data reported here is presented as a % of the contralateral or non-ischemic hemisphere. Often, MCAO experiments undertaking LSCI utilise continuous imaging pre and post-MCAO, preventing variability in animal/camera placement, focus level and instrument settings, improving the validity of obtained data and allowing within experiment data normalisation/analysis. Due to the experimental design of this study, data was normalised within experiment to the non-stroke hemisphere, as reported previously ^33– 36^, to reduce the impact of between experiment variability from, for example equipment setup. However, it is important to note that this approach does not take into account the dynamic transhemispheric effect on CBF shown during focal ischemia reperfusion ^37^. In addition to the expected CBF variations during and following MCAO, CBF in the contralateral hemisphere has also been reported to be affected during this pathology. The contralateral hemisphere is an important reserve to balance blood flow along the ischemic gradient to the ipsilateral hemisphere via collateral flow, particularly where reperfusion is reliant on flow from the basilar artery and contralateral CCA ^18^. It is also worthy to note that a sustained reduction in CBF measurements within the CCA ligated group is clear. This hypoperfusion could lead to exacerbation of post-stroke outcomes, with reduced oxygen levels resulting in pathological changes such as inflammation, demyelination and apoptosis ^38^. Chronic sub-ischemic hypoperfusion is a key characteristic of conditions such as vascular dementia and there is a strong clinical association between hypoperfusion and development of vascular dementia ^39^. In a further study it would be interesting to assess the impact of improved reperfusion on brain injury/recovery and functional outcome associated with ischemia.

In summary, this brief report shows that CCA repair following MCAO, in the mouse, using a homologous tissue pad combined with sealant improves blood flow to the ischemic hemisphere. The results validate the use of CCA repair in MCAO studies to remove reliance on the CoW avoiding the variation associated with this structure, in a commonly used mouse strain, on post-stroke measures. The outcome reported here may be of particular interest for use in endovascular thrombectomy research, mimicking the clinical situation of clot removal without ligation of arteries.

## Data availability

### Underlying data

Zenodo: Dataset corresponding to scientific paper “Improved reperfusion following alternative surgical approach for experimental stroke in mice”. https://doi.org/10.5281/zenodo.3701231 ^40^.

This project contains the following underlying data:

- BASE_Repair (baseline raw LCSI export files for animals that underwent repair of the CCA; the three-symbol code at the start of each file represents a different mouse).
- 24h_Repair (24-hour raw LCSI export files for animals that underwent repair of the CCA; the three-symbol code at the start of each file represents a different mouse).
- 48h_Repair (48-hour raw LCSI export files for animals that underwent repair of the CCA; the three-symbol code at the start of each file represents a different mouse).
- BASE_Ligated (baseline raw LCSI export files for animals that underwent ligation of the CCA; the three-symbol code at the start of each file represents a different mouse).
- 24h_Ligated (24-hour raw LCSI export files for animals that underwent ligation of the CCA; the three-symbol code at the start of each file represents a different mouse).
- 48h_Ligated (48-hour raw LCSI export files for animals that underwent ligation of the CCA; the three-symbol code at the start of each file represents a different mouse).
- 20200206_F1000_LS_Manuscript Raw Data (LCSI Flux values obtained by drawing ROIs in the LSCI software and exporting the values).

Data are available under the terms of the Creative Commons Attribution 4.0 International license (CC-BY 4.0)
