# Supplementary material for: Improved reperfusion following alternative surgical approach for experimental stroke in mice
Source: F1000Res. 2020 May 13;9:188. Originally published 2020 Mar 13. [Version 3] doi: 10.12688/f1000research.22594.3 (PMC7217225; doi:10.12688/f1000research.22594.3)
Supplement: Peer review history

# Peer review: Reviewer response for version 3

**DOI:** 10.5256/f1000research.26544.r63375

**Published:** 2020-05-14

**Authors:** Brad Sutherland

**Affiliations:** 1 School of Medicine, College of Health and Medicine, University of Tasmania, Hobart, Tas, Australia

Thanks for the change. The concluding paragraph reads more clearly now.

Is the work clearly and accurately presented and does it cite the current literature?

Yes

If applicable, is the statistical analysis and its interpretation appropriate?

Yes

Are all the source data underlying the results available to ensure full reproducibility?

Yes

Is the study design appropriate and is the work technically sound?

Yes

Are the conclusions drawn adequately supported by the results?

Yes

Are sufficient details of methods and analysis provided to allow replication by others?

Yes

Reviewer Expertise:

Stroke and cerebral blood flow

I confirm that I have read this submission and believe that I have an appropriate level of expertise to confirm that it is of an acceptable scientific standard.

# Peer review: Reviewer response for version 2

**DOI:** 10.5256/f1000research.26258.r63085

**Published:** 2020-05-11

**Authors:** Brad Sutherland

**Affiliations:** 1 School of Medicine, College of Health and Medicine, University of Tasmania, Hobart, Tas, Australia

Thank you to the authors for their detailed response. The manuscript has improved and my comments have all been adequately addressed. The only minor point is the request to change the second last sentence of the discussion "Validating the use of CCA repair..." does not seem to have changed, and this sentence still reads as an incomplete sentence. However, this is very minor and should not prevent acceptance.

Is the work clearly and accurately presented and does it cite the current literature?

Yes

If applicable, is the statistical analysis and its interpretation appropriate?

Yes

Are all the source data underlying the results available to ensure full reproducibility?

Yes

Is the study design appropriate and is the work technically sound?

Yes

Are the conclusions drawn adequately supported by the results?

Yes

Are sufficient details of methods and analysis provided to allow replication by others?

Yes

Reviewer Expertise:

Stroke and cerebral blood flow

I confirm that I have read this submission and believe that I have an appropriate level of expertise to confirm that it is of an acceptable scientific standard.

# Response

**Published:** 2020-05-11

**Authors:** claire gibson

**Affiliations:** university of nottingham, UK

1. The only minor point is the request to change the second last sentence of the discussion "Validating the use of CCA repair..." does not seem to have changed, and this sentence still reads as an incomplete sentence. 

 Response: This edit had been missed from the final edit of version 2. The concluding paragraph has been rewritten to improve clarity and address the sentence structure.

# Peer review: Reviewer response for version 1

**DOI:** 10.5256/f1000research.24941.r61345

**Published:** 2020-04-07

**Authors:** Brad Sutherland

**Affiliations:** 1 School of Medicine, College of Health and Medicine, University of Tasmania, Hobart, Tas, Australia

This manuscript by Trotman-Lucas is a brief report outlining a technical refinement of the intraluminal filament middle cerebral artery occlusion model that leads to improved reperfusion. The authors have previously shown that CCA repair can increase acute CBF during reperfusion and consequently have reduced infarct volume size and variability. This manuscript provides a nice addition with Laser Speckle Contrast Imaging showing reperfusion changes lasting 48 hours between the traditional Koizumi method and the CCA repair method. This simple repair method could improve our ability to conduct stroke modelling. I have some comments below.

 Specific comments:

1. The Laser Speckle Contrast Imaging method has great spatial and temporal resolution but does not provide any depth assessment of CBF changes in the brain. It only provides a measurement of CBF on the surface of the brain. Therefore, all that can be stated from this study is that pial CBF can be improved post-MCA during CCA repair compared to the CCA ligated method, as we do not know from this imaging technique whether the underlying brain is fully reperfused. Some discussion around this would be useful.
2. The Laser Speckle Contrast Imaging was conducted at baseline (prior to MCAO), 24h post-MCAO and 48h post-MCAO. However, there was no apparent assessment during MCAO to show that a stroke had actually occurred. How can you confirm that these animals actually had a stroke with no CBF assessment during MCAO?
3. The sustained (48h) changes shown in Figure 1 are interesting. It would have been excellent to have shown how these CBF values are associated with changes in neurological deficit (could be done by a neuroscore) or infarct volume at 48 hours. Were the brains assessed for this?
4. The CCA ligated results show sustained hypoperfusion on the surface of the brain for 48 hours. Some extended discussion on how this could impact the development of the lesion and chronic injury would be useful, particularly since chronic occlusion of the CCA is used in vascular dementia studies with chronic neurodegeneration and impaired cognition present.
5. There are many studies showing that CBF in the contralateral hemisphere can be altered during MCAO and during reperfusion (e.g. Lavy et al 1975 Stroke 6:160-3 ^1^; Taheri et al 2019 Transl Neurosci Res Rev 2:27-37 ^2^; Premilovac et al 2020 JCBFM 271678X20905493 ^3^ and others). Given that the quantification of the ipsilateral CBF is a % of the contralateral, any changes in CBF in the contralateral hemisphere in response to MCAO or reperfusion could alter the reference point for this analysis. Some discussion should be added about this as a limitation.
6. The second last sentence of the discussion “Validating the use of CCA repair…” is an incomplete sentence and will need rewritten.

Is the work clearly and accurately presented and does it cite the current literature?

Yes

If applicable, is the statistical analysis and its interpretation appropriate?

Yes

Are all the source data underlying the results available to ensure full reproducibility?

Yes

Is the study design appropriate and is the work technically sound?

Yes

Are the conclusions drawn adequately supported by the results?

Yes

Are sufficient details of methods and analysis provided to allow replication by others?

Yes

Reviewer Expertise:

Stroke and cerebral blood flow

I confirm that I have read this submission and believe that I have an appropriate level of expertise to confirm that it is of an acceptable scientific standard, however I have significant reservations, as outlined above.

## References

[1] : The effect of cerebral infarction on the regional cerebral blood flow of the contralateral hemisphere. *Stroke*.6(2) : 10.1161/01.str.6.2.160 160-3 10.1161/01.str.6.2.160 1121715

[2] : Transhemispheric Diaschisis after Unilateral Focal Cerebral Ischemia Reperfusion: A Longitudinal Voxel-Based Study by MRI. *Transl Neurosci Res Rev*.2019;2(1) : 10.36959/817/523 10.36959/817/523

[3] : Transcranial contrast-enhanced ultrasound in the rat brain reveals substantial hyperperfusion acutely post-stroke. *J Cereb Blood Flow Metab*.2020; 10.1177/0271678X20905493 271678X20905493 10.1177/0271678X20905493 32063081PMC7181087

# Response

**Published:** 2020-04-29

**Authors:** claire gibson

**Affiliations:** university of nottingham, UK

1. The Laser Speckle Contrast Imaging method has great spatial and temporal resolution but does not provide any depth assessment of CBF changes in the brain. It only provides a measurement of CBF on the surface of the brain. Therefore, all that can be stated from this study is that pial CBF can be improved post-MCA during CCA repair compared to the CCA ligated method, as we do not know from this imaging technique whether the underlying brain is fully reperfused. Some discussion around this would be useful.

Response: Please see added section into discussion.

  

1. The Laser Speckle Contrast Imaging was conducted at baseline (prior to MCAO), 24h post-MCAO and 48h post-MCAO. However, there was no apparent assessment during MCAO to show that a stroke had actually occurred. How can you confirm that these animals actually had a stroke with no CBF assessment during MCAO?

Response: As now stated in the methods section of the paper the drop in CBF during MCAO was confirmed using laser doppler flowmetry.

  

1. The sustained (48h) changes shown in Figure 1 are interesting. It would have been excellent to have shown how these CBF values are associated with changes in neurological deficit (could be done by a neuroscore) or infarct volume at 48 hours. Were the brains assessed for this?

Response: We would agree that any further analysis under these experimental conditions would be useful.  However, that was beyond the remit of this current study which was powered to answer the specific research question.  We have previously reported the effect of this modified surgical approach on lesion volume (see Trotman et al., 2017, Disease Models & Mechanisms).

  

1. The CCA ligated results show sustained hypoperfusion on the surface of the brain for 48 hours. Some extended discussion on how this could impact the development of the lesion and chronic injury would be useful, particularly since chronic occlusion of the CCA is used in vascular dementia studies with chronic neurodegeneration and impaired cognition present.

Response: Please see amended section in discussion.

  

1. There are many studies showing that CBF in the contralateral hemisphere can be altered during MCAO and during reperfusion (e.g. Lavy et al 1975 Stroke 6:160-3 ^1^; Taheri et al 2019 Transl Neurosci Res Rev 2:27-37 ^2^; Premilovac et al 2020 JCBFM 271678X20905493 ^3^ and others). Given that the quantification of the ipsilateral CBF is a % of the contralateral, any changes in CBF in the contralateral hemisphere in response to MCAO or reperfusion could alter the reference point for this analysis. Some discussion should be added about this as a limitation.

Response: Please see amended section in discussion.

  

1. The second last sentence of the discussion “Validating the use of CCA repair…” is an incomplete sentence and will need rewritten.

Response: This sentence has been amended.

# Peer review: Reviewer response for version 1

**DOI:** 10.5256/f1000research.24941.r61343

**Published:** 2020-03-30

**Authors:** Arshad Majid

**Affiliations:** 1 Sheffield Institute for Translational Neuroscience, University of Sheffield, Sheffield, UK

This is a well written concise study on refinement of the MCAO model in mice. The investigators show that repair of the CCA achieves better reperfusion of the hemisphere than ligation of the CCA This article is a useful addition to the literature. 

 It would be useful to know how stable the underlying infarction is using this model. These data are not shown.

Is the work clearly and accurately presented and does it cite the current literature?

Yes

If applicable, is the statistical analysis and its interpretation appropriate?

Yes

Are all the source data underlying the results available to ensure full reproducibility?

Yes

Is the study design appropriate and is the work technically sound?

Yes

Are the conclusions drawn adequately supported by the results?

Yes

Are sufficient details of methods and analysis provided to allow replication by others?

Yes

Reviewer Expertise:

Preclinical models of stroke

I confirm that I have read this submission and believe that I have an appropriate level of expertise to confirm that it is of an acceptable scientific standard.

# Response

**Published:** 2020-03-30

**Authors:** claire gibson

**Affiliations:** university of nottingham, UK

Thank you for this review.  In response to the specific point about lesion volume - we did not investigate that within this study as we have previously (Trotman-Lucas M, Kelly ME, Janus J, *et al.*: An alternative surgical approach reduces variability following filament induction of experimental stroke in mice. *Dis Model Mech.* 2017; 10(7): 931–938) demonstrated the impact of this refined surgical approach on lesion volume.

# Response

**Published:** 2020-05-05

**Authors:** Arshad Majid

**Affiliations:** University of Sheffield, UK

Thank you for your response.
